# MicroRNA-126-3p suppresses cell proliferation by targeting PIK3R2 in Kaposi's sarcoma cells

**DOI:** 10.18632/oncotarget.9311

**Published:** 2016-05-12

**Authors:** Xiu-Juan Wu, Zong-Feng Zhao, Xiao-Jing Kang, Hong-Juan Wang, Juan Zhao, Xiong-Ming Pu

**Affiliations:** ^1^ Department of Dermatology and Venereology, People's Hospital of Xinjiang, Uygur Autonomous Region, Urumqi, Xinjiang, China; ^2^ Clinical Medical Research Center, People's Hospital of Xinjiang, Uygur Autonomous Region, Urumqi, Xinjiang, China

**Keywords:** miR-126-3p, suppress, target, PIK3R2, Kaposi's sarcoma

## Abstract

Kaposi's sarcoma is a highly vascular tumor of lymphatic endothelial origin. Many deregulated miRNAs, including miR-126-3p, have been identified in Kaposi's sarcoma tissues. miR-126-3p is the most highly endothelial-specific miRNA that regulates vascular integrity and angiogenesis. In this study, we aimed to determine the effect of miR-126-3p on Kaposi's sarcoma cells through transfection of a miRNA mimic and inhibitor. Moreover, we searched the target gene (*PIK3R2*) of miR-126-3p using bioinformatics software and further verified PIK3R2 using luciferase reporter assays, Real-time quantitative PCR (qRT-PCR) and western blot. The results demonstrated that miR-126-3p inhibited cell proliferation, arrested cell cycle progression, induced cell apoptosis, and inhibited cell invasion of SLK cells. The bioinformatics analysis and luciferase reporter assay revealed that *PIK3R2* mRNA is a direct target of miR-126-3p. Moreover, the level of expression of the *PIK3R2* gene was downregulated in SLK cells transfected with miR-126-3p siRNAs. Therefore, our data demonstrated that miR-126-3p is a tumor suppressor miRNA that acts by targeting *PIK3R2* in Kaposi's sarcoma cells. These findings contribute to our understanding of the molecular mechanisms underlying Kaposi's sarcoma.

## INTRODUCTION

Kaposi's sarcoma (KS) is a multicentric tumor of mesenchymal origin which was first described by Moritz Kaposi in 1872 [1, 2]. It mostly affects elderly men of Italian, Jewish, or Mediterranean origin [3]. In China, more than 90 % of KS cases, including classic KS (CKS) and AIDS-associated KS (AIDS-KS), have occurred in Xinjiang, especially among Uyghur patients [4–6]. Many studies have demonstrated that human herpes virus 8 (HHV-8) is an important pathogen in KS, and more than 95% of patients with KS have been infected with HHV-8 [7]. However, the pathogenesis of KS remains unclear.

In recent years, miRNAs have become known to play important roles in cancer; they are involved in the etiology, diagnosis, treatment and prognosis [8, 9]. miRNAs are a class of small noncoding RNAs that silence target genes expression by binding to the 3′-untranslated regions (3′UTRs) of miRNAs. miRNAs can degrade target mRNAs or inhibit their translation [10]. One study showed that miR-143/145 is an upregulated miRNA biomarker and that miR-221/222, miR-155, and the let-7 family are downregulated in KS [11]. Wu et al. [12] clearly identified 170 deregulated miRNAs: 69 were upregulated and 101 downregulated when compared between KS and matched adjacent healthy tissues. In particular, miR-126-3p and the 13 KSHV-related miRNAs were upregulated. Another study showed that there were 185 differentially expressed miRNAs, of which 76 were upregulated and 109 were downregulated, in 17 formalin-fixed paraffin-embedded KS samples and three Kaposi's sarcoma associated herpesvirus (KSHV) -negative normal Formalin fixed paraffin embedding (FFPE) samples [13]. These reports reveal that deregulated miRNAs are involved in the occurrence and development of KS.

The miR-126-3p is an intronic miRNA, located in intron 7 of the epidermal growth factor-like protein 7 gene (EGFL7) on chromosome 9. miR-126-3p is a highly conserved gene whose product is located in the endothelial cells of blood vessels and regulates angiogenesis and vasculogenesis [14]. Knockout of miR-126-3p can cause a loss in blood vessel integrity and result in hemorrhage during embryonic development in zebrafish, showing that miR-126-3p controls blood vessel integrity and angiogenesis [15]. Many studies have suggested that miR-126-3p plays a part as either an anti-oncogene or an oncogene in different cancers [16, 17]. miR-126-3p also acts as a tumor suppressor via the downregulation of intron 7 of epidermal growth factor-like domain 7 (EGFL7) and it may be a promising candidate for therapeutic strategies in oral squamous cell carcinoma [18]. Some studies have demonstrated that miR-126-3p stimulates angiogenesis by targeting PIK3R2 proteins, which are negative regulators of the vascular endothelial growth factor (VEGF) signal pathway [19].

However, the functions and molecular mechanism of miR-126-3p in KS remain unclear. In this study, we aimed to investigate the roles of miR-126-3p in KS cells (SLK cells) using transfection of a mimic and inhibitor. Furthermore, we showed that miR-126-3p regulates its target PIK3R2 and further explored the molecular mechanisms underlying KS.

## RESULTS

### miR-126-3p inhibits cell proliferation in SLK cells

**Figure 1:**
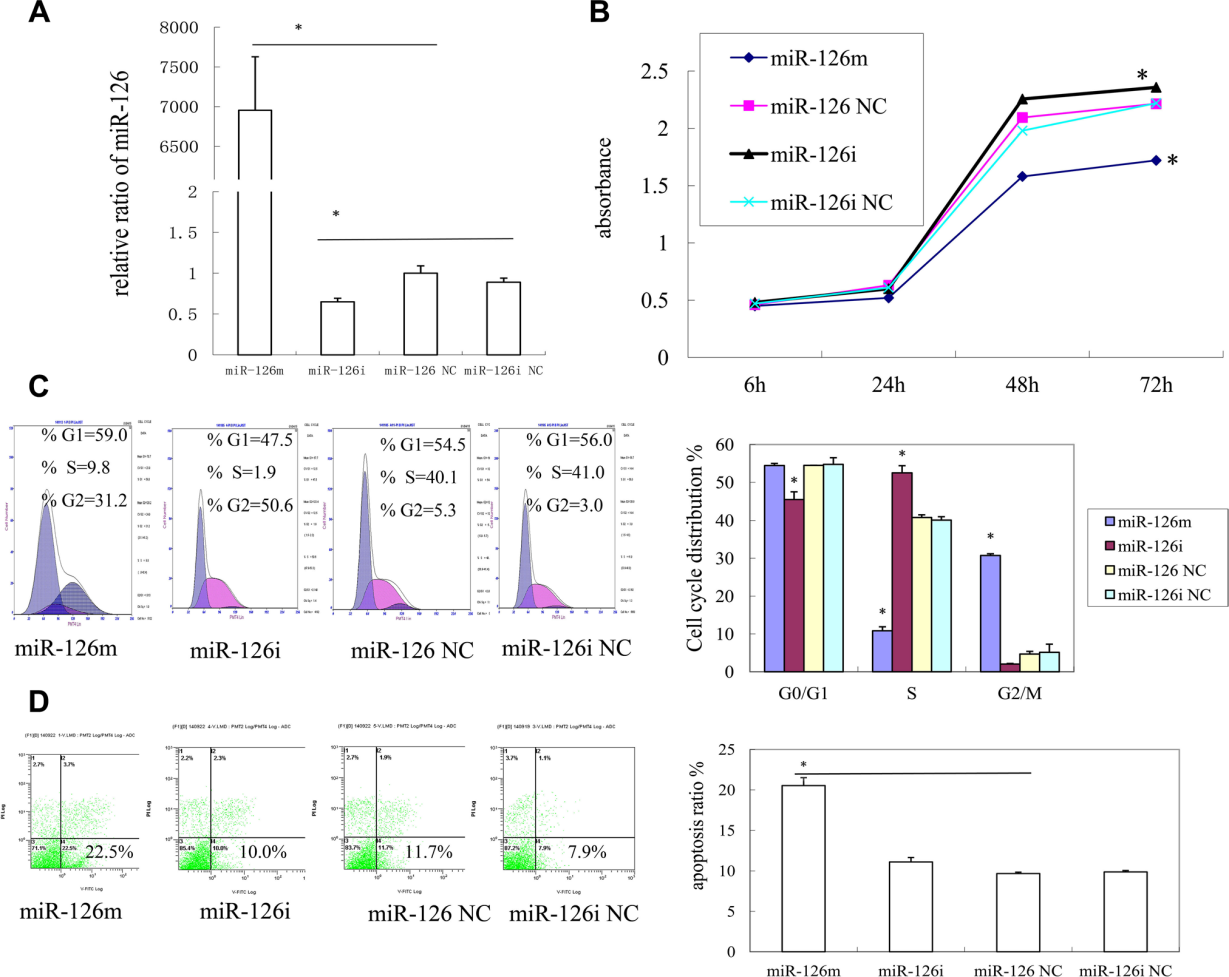
The effects of miR-126 on KS cell proliferation (**A**) The expression of miR-126 in SLK cell after transfection mimics, inhibitor, negative control and inhibitor negative control for 48 h by qRT-PCR (**B**) Cell proliferation assay of SLK cells was performed after transfection with miR-126 mimics, inhibitor, NC, or inhibitor NC by using CCK-8.(**C**) cell cycle analysis of SLK cells after transfection was performed by PI staining. MiR-126 arrested cell cycle in G2 phase. (**D**) The effects of miR-126 on KS cell apoptosis. Cell apoptosis of SLK cells upon transfection with miR-126 mimics, inhibitor, NC, or inhibitor NC was detected bv PE Annexin-V staining.

We demonstrated the functional role of miR-126-3p in the proliferation of SLK cells after transfection with miR-126-3p mimics, inhibitor, negative control and inhibitor negative control. The transfection efficiency of SLK cells was assayed at 48 h post-transfection (Figure 1A). Cell proliferation was measured using a CCK-8 kit. Compared with the negative control, SLK cells transfected with miR-126-3p mimics showed lower cell growth viability. In contrast, SLK cells transfected with the miR-126-3p inhibitor exhibited greater cell growth than those transfected with the inhibitor negative control. Therefore, we found that miR-126-3p may inhibit cell growth (Figure 1B).

### miR-126-3p induces cell cycle arrest at the G2/M phase

Cell cycle analyses showed that SLK cells transfected with miR-126-3p mimics had an obvious increase in the G2/M phase, when compared with negative control cells. In contrast, fewer SLK cells transfected with an inhibitor were in the G2/M phase when compared with those transfected with the inhibitor negative control. These results showed that miR-126-3p arrests the cell cycle in the G2/M phase and therefore interrupts DNA synthesis and cell proliferation (Figure 1C).

### miR-126-3p promotes cell apoptosis

We also investigated cell apoptosis using PE Annexin-V stain. As shown in Figure 1D, SLK cells transfected with miR-126-3p mimics had an increased proportion in early apoptosis, when compared with those transfected with negative control. Therefore, miR-126-3p induced apoptosis of SLK cells.

### miR-126-3p inhibits invasion by SLK cells

**Figure 2:**
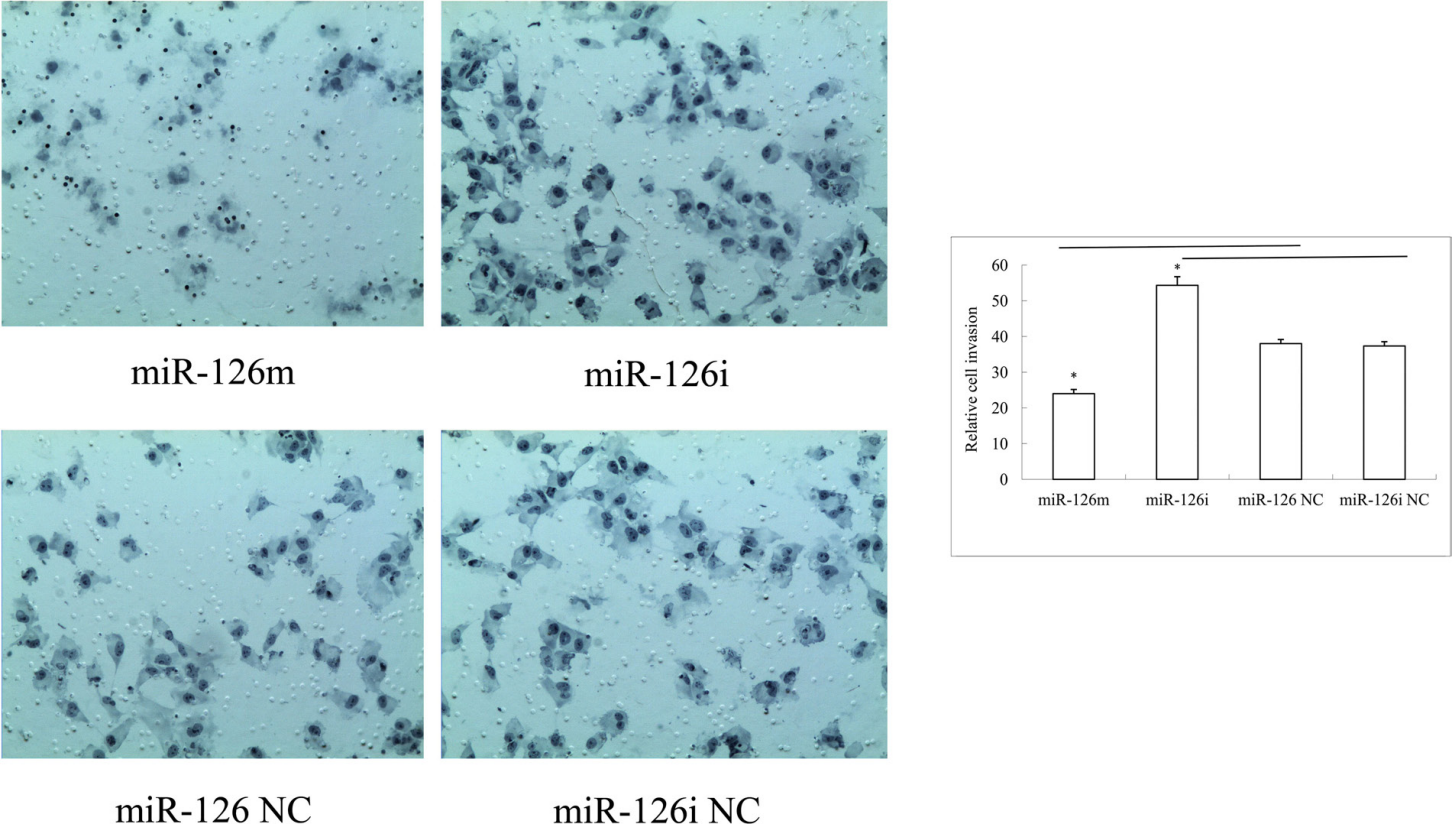
Representative photos and statistical plots of transwell assays in SLK cells transfected with miR-126-3p mimics, miR-126-3p NC, miR-126-3p inhibitor and inhibitor negative control More cells traversed the transwell membrane in miR-126-3p inhibitor transfected cells, and fewer cells in the miR-126-3p mimics transfected cells.

The cell invasion assay demonstrated that more cells traversed the transwell membrane among miR-126-3p inhibitor transfected cells, and fewer cells among the miR-126-3p mimic transfected cells. The SLK cells transfected with miR-126-3p mimics had a lower invasion rate than with miR-126 negative control (Figure 2). Compared with an inhibitor negative control, transfection of the SLK cell line with the miR-126-3p inhibitor also increased invasive ability (Figure 2).

### miR-126-3p directly targets PIK3R2

**Figure 3:**
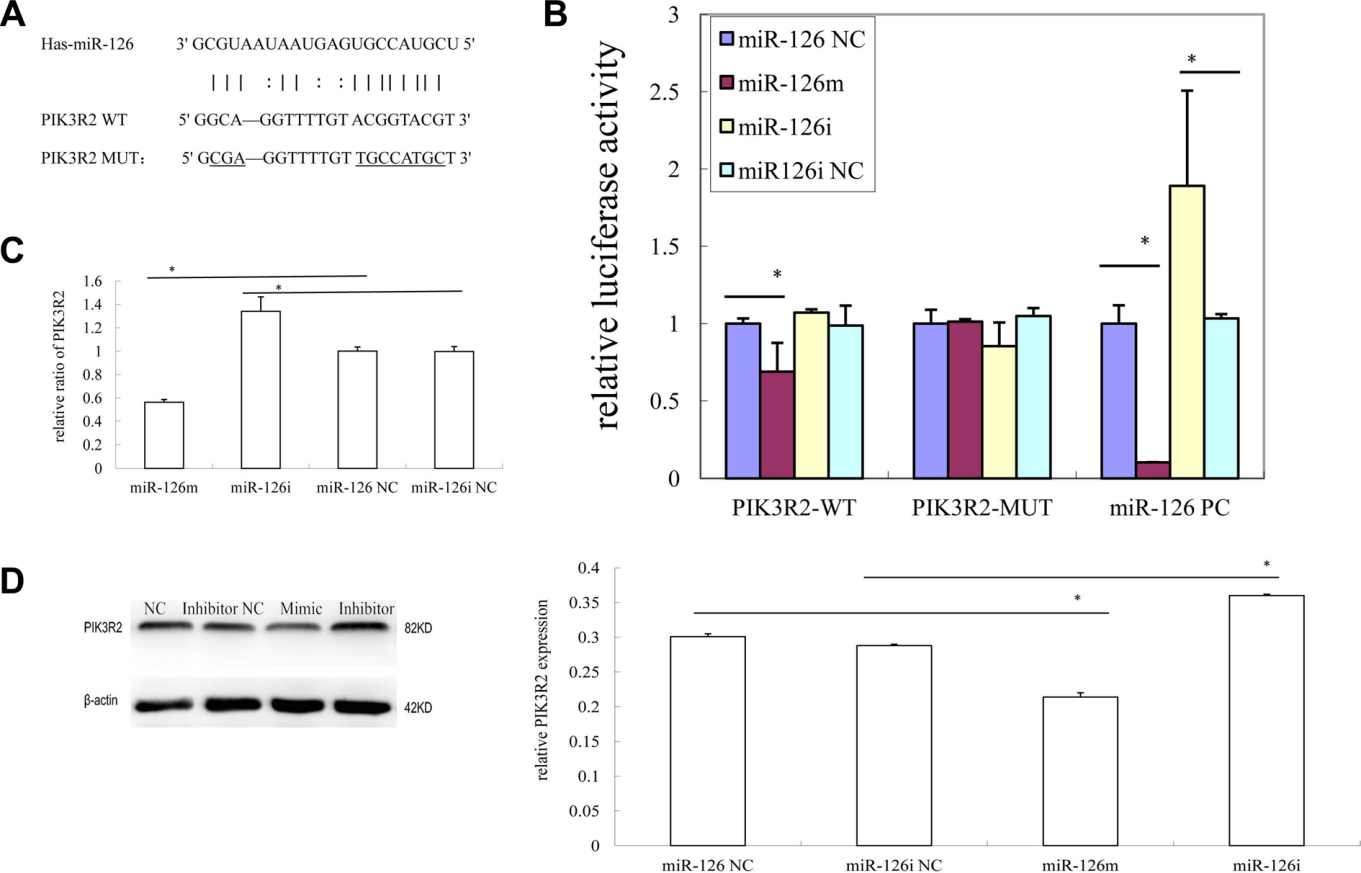
MiR-126 targets PIK3R2 gene in KS cells (**A**) Schematic representation of PIK3R2 3′UTR showing putative miRNA target site; (**B**) The indicated PIK3R2 reporter construct in SLK cells, co-transfected with miR-126 mimics, inhibitor, NC, or inhibitor NC, was detected using relative luciferase activity. (**C**) Quantitative RT-PCR assay was performed to detect the expression of PIK3R2 upon transfection with miR-126 mimics, inhibitor, NC, or inhibitor NC; (**D**) Western blot analysis of the expression of PIK3R2 protein in SLK cells transfected with miR-126 mimics, inhibitor, NC, or inhibitor NC was performed.

The programs miRBase, miRanda, and Target Scan were used to predict putative target genes in 3′UTR binding sites of miR-126-3p. Given its involvement in apoptosis, and in the mTOR and PI3K-Akt signaling pathways, we selected *PIK3R2* as the possible target gene of miR-126-3p. We synthesized target sequences of *PIK3R2*-wild type and PIK3R2-mutant type (Figure 3A). Subsequently, we cloned the 3′UTRs of PIK3R2-wild type and PIK3R2-mutant type into the pmirGLO plasmid and investigated, by the dual-luciferase reporter assay, whether miR-126-3p can inhibit the expression of PIK3R2. The results showed that miR-126-3p inhibited the level of cell activity of the PIK3R2-wild type. Conversely, miR-126-3p did not suppress the level of cell activity of the PIK3R2-mutant type (Figure 3B).

We also assayed the level of expression of the PIK3R2 genes, using both qRT-PCR and western blot analysis, in SLK cells transfected with miR-126-3p siRNAs. With overexpression of miR-126-3p, the level of expression of PIK3R2 mRNA and PIK3R2 protein showed a significant reduction. In contrast, inhibition of miR-126-3p caused an obvious increase in the expression of PIK3R2 mRNA and in PIK3R2 protein (Figure 3C and 3D).

## DISCUSSION

KS is a highly vascular tumor of endothelial lymphatic origin. It is characterized by the presence of proliferating spindle-shaped tumor cells, infiltration of inflammatory cells and fibrosis, extravasation of erythrocytes and hemosiderin storage, and enhanced neovascularization [20]. However, little is known about the underlying molecular basis of KS. Recently, deregulated expression of some miRNAs has been reported [11–13]; miRNAs are endogenous non-coding RNA molecules, 18–25 nucleotides (nt) in length, that negatively regulate downstream target genes. They play important functions in development, cell differentiation, and regulation of the cell cycle and apoptosis [21]. miRNAs can act as oncogenes and tumor suppressors associated with deregulated gene expression in cancer caused by gene amplification, deletion, mutation, and epigenetic silencing. miRNAs can also guide the diagnosis, prognosis, and treatment of cancer [22, 23]. miR-126-3p inhibits cell migration, and is associated with reorganization of the cytoskeleton, vascular integrity, endothelial phenotype and cell survival *in vitro* and *in vivo* [15].

In this study, we studied miR-126-3p by transfection of a miR-126-3p mimic and inhibitor into the SLK cell line using cytological methods *in vitro*. The results indicated that miR-126-3p inhibited cell proliferation, arrested cell cycle progression, induced cell apoptosis and inhibited cell invasion. These data showed that miR-126-3p has a tumor suppressor role in KS. Expression of miR-126-3p was reported to be downregulated in hepatocellular carcinoma, and to have a tumor suppressor role associated with inhibition of cell proliferation, prevention of cell cycle progression and induction of cell apoptosis in a hepatocellular carcinoma cell line [24]. miR-126-3p is expressed specifically in endothelial cells and has important effects in vasculogenesis, angiogenesis, and tumor growth. miR-126-3p is involved with the VEGF/PI3K/AKT signaling pathway and targets both VEGFA and PIK3R2, playing a role in vasculogenesis and tumor growth in human breast cancer [25]. Some studies have revealed that miR-126-3p inhibits cancer growth directly by targeting Sox2, p85β (PIK3R2), IRS1, VEGF and other genes [24, 26–28].

We chose to study PIK3R2, among hundreds of genes, because it is closely involved in cell migration, proliferation, and survival [29, 30]. Dual-luciferase reporter assays showed that the cotransfection of miR-126-3p and PIK3R2-WT induced a substantial reduction of luciferase activity. Ectopically expressed miR-126-3p also reduced the level of expression of the mRNA and protein of PIK3R2, which demonstrated the direct role of PIK3R2 as a target in SLK cells. PI3K is a complex composed of regulatory subunits (p85α, p85β, and p85γ) and catalytic subunits (p110), and acts as a pivotal growth factor signal. p85β, encoded by the PIK3R2 gene, is an enzyme that generates 3-polyphosphoinositides at the plasma membrane, inhibits phosphorylation of Akt and activates PI3K. The deletion of PIK3R2 increases insulin-induced Akt activation, leading to increased anti-apoptosis [31]. Furthermore, shRNA suppression of PIK3R2 induces activation of PI3K/Akt [32]. However, PIK3R2 has been shown to be an oncogene in breast and colon cancer and its induced overexpression of PIK3R2 correlates with PI3K pathway activation and tumor progression *in vivo* [33]. miR-126-3p inhibits cancer growth via directly targeting Sox2 and various other genes. Moreover, in addition to p85β (PIK3R2) and Sox2, IRS1, VEGF and CXCR4 have been reported to be target genes of miR-126-3p and to participate in miR-126-3p-induced tumor suppression [17, 24, 27].

In conclusion, our results have demonstrated that miR-126-3p can inhibit cell growth, arrest cell cycle progression, induce cell apoptosis, inhibit cell invasion and downregulate the level of expression of PIK3R2 in SLK cells. miR-126-3p is a tumor suppressor miRNA that acts by targeting PIK3R2 in KS cells. These findings contribute to our understanding of the molecular mechanism of KS and provide a strong foundation for further investigation of the impact of PIK3R2 in KS.

## MATERIALS AND METHODS

### Cell line

The human KS-derived SLK cell line, obtained from NIH AIDS Reagent Program [34], was cultured in RPMI 1640 medium (Gibco, Grand Island, NY, USA) supplemented with 10% fetal bovine serum (Gibco, Grand Island, NY, USA) in a humidified atmosphere of 5% CO_2_ and 95% air at 37°C.

### Identification of miRNA target gene

The miRBase (http://www.mirbase.org), miRanda (http://www.microrna.org/), and TargetScan (http://www.targetscan.org/vert_61/) programs were used to predict putative miRNAs binding sites in the 3′UTR of human PIK3R2 (NM_005027).

### Transfection of miR-126-3p mimic and inhibitor in SLK cells

The miR-126-3p mimic (miR-126m, Product ID:219600), miR-126-3p inhibitor (miR-126i, Product ID:219300), miScript Inhibitor Negative Control miR-126-3p (miR-126iNC, Product ID:1027271) and AllStars Negative control siRNA (miR-126 NC, Product ID:1027280) were purchased from Qiagen (Qiagen, Hilden, Germany) and transfected into cells using HiPerFect Transfection Reagent (Product ID:301704, Qiagen, Hilden, Germany) as performed by the manufacturer.

### Quantitative real-time reverse transcriptase PCR (qRT-PCR)

For cultured cells, the total RNA was isolated from SLK cells using QIAzol Lysis Reagent (Qiagen) and reverse transcribed with the miScript II Reverse-Transcription Kit (Qiagen) according to the manufacturer's instructions. RNA concentrations were measured using a Nanodrop spectrophotometer (ND-1000, Germany), and RNA integrity was determined by gel electrophoresis. The levels of expression of miR-126-3p and PIK3R2 were measured by qRT-PCR with an miScript SYBR Green PCR Kit (Qiagen) in a Qiagen Roter-Gene Q. The primers used for the detection of miR-126-3p, U6, PIK3R2 and β-actin were the Hs_miR-126 miScript Primer Assay (MS00003430, Qiagen), the Hs_RNU6 miScript Primer Assay (MS00033740, Qiagen), the Hs_PIK3R2 Primer Assay (QT01006005, Qiagen) and the Hs_β-actin Primer Assay (QT00095431, Qiagen), respectively. All reactions were performed in triplicate. The relative expression level was calculated by using the 2^−ΔΔCt^ analysis method.

### Cell proliferation assay

Cells were transfected with 10 nM miRNA/miRNA inhibitor by fast-forward transfection and plated at a final concentration of 2 × 10^3^ cells per well in 96-well plates. The proliferation rate was evaluated using a Cell Counting Kit-8 (CCK-8, Saichi, Beijing) at 6, 24, 48 and 72 h after transfection. The optical density at 570 nm (OD570) of each well was measured with an enzyme-linked immunosorbent assay (ELISA) reader (Thermo scientific, US). All experiments were repeated three times in triplicate.

### Cell cycle assay

The cells were digested with trypsin and collected after transfection for 48 h. Cells were washed twice with cold PBS, resuspended in PBS and then fixed at −20°C for 1 h in 75% ethanol. The cells were washed with cold PBS and incubated with 500 ng/μl of RNase A at 37°C for 30 min and then stained with 400 μl propidium iodide at 4°C for 30 min. The stained cells (1.5 × 10^5^) were analyzed with a flow cytometer (BD Biosciences, San Jose, CA, USA). Experiments were performed in triplicate.

### Cell apoptosis assay

The cells were collected after transfection for 48 h and detected by analyzing Annexin V-FLOUS Staining kit binding by flow cytometry using a FITC signal detector and a propidium iodide (PI) signal detector.

### Cell invasion assay

Cell invasion was investigated using a transwell chamber assay coated with Matrigel (Corning, NY, USA) according to the manufacturer's instruction. The SLK cells were seeded on an 8-μm pore size transwell insert coated with extracellular matrix (ECM) for the invasion assay. After incubation at 37°C for 48 h, we adjusted the cell density to 2 × 10^4^/ml. We added 200 μl of a 2 × 10^4^/ml single cell suspension to each transwell chamber, and the transwell chambers were incubated at 37°C for 24 h. Using a cotton applicator, the cells adherent to the upper surface of the filter were removed, and then stained with hematoxylin. The number of cells that had passed through the pores into the lower chamber was counted under a phase-contrast microscope.

### Western blot

The cells were harvested in cold PBS after 48 h transfection and prepared by lysis in Regulation Of Investigatory Powers Act (RIPA) buffer with protease inhibitors at 4°C for 30 min. The protein concentration of the samples was measured using a BCA Protein Assay Kit (Tiangen). Proteins were separated on a 10% separation gel and 5% spacer gel and transferred to PVDF membranes. The membrane was blocked with 5% bovine serum albumin (BSA) at room temperature for 1 h and washed three times with TBST. The membrane was incubated with primary PIK3R2 antibody (Abcam, USA) diluted 1:200, at 4°C overnight. On the following day, the membrane was washed three times with TBST and incubated with HRP-conjugated secondary antibody (Abcam) diluted 1:10,000 with TBST at room temperature for 1 h. Subsequently, 1 ml of mixed colored liquids A and B was added to the membrane and detected using a ChemiScope 3000 chemiluminescence instrument to calculate the optical density of the target protein with reference to β-actin levels.

### Plasmid construction, transfection and luciferase reporter assays

To generate the luciferase reporter plasmid, the wild-type or mutant PIK3R2 3′-UTR were amplified from genomic DNA and cloned into pmirGlO vectors (Promega, Wisconsin, USA) using the following primers: PIK3R2 wild-type 3′UTR forward primer, 5′-CCACGAGCTGGGAGGCAGGTTTTGTACGGTACGTTGTTATTG ATATG ATATAAAACATCAAC-3′, reverse primer, 5′-TCG AGTTGATGTTTTATATCATAT CAATAACAACGTAC CGTACAAAACCTGCCTCCCAGCTCGTGGAGCT-3′. PIK3R2 mutant-type 3′UTR forward primer, 5′-CCAC GAGCTGGGAGGCAGGTTTT GTTGCCATGCTTGTT ATTGATATGATATAAAACATCAAC -3′, reverse primer, 5′- TCGAGTTGATGTTTTATATCATATCAATAACAA GCATGGCAACAAAACCTGCCTCCCAGCTCGTGGA GCT -3′. About 1 × 10^5^ 293T cells per well were seeded into 24-well plates for 24 h before transfection. The cells were co-transfected with 0.8 μg wild-type or mutant PIK3R2 pmirGlO luciferase reporter and 40 nM miR-126-3p mimic or miR-126-3p inhibitor using Lipofectamine 2000 (Invitrogen, Carlsbad, CA, USA). A luciferase reporter construct containing the miR-126-3p consensus target sequence served as the positive control (PC) and the PmirGLO vector served as the internal control. At 48 h post-transfection, cells were assayed for luciferase activity using the Dual-Luciferase Reporter Assay (Promega) according to the manufacturer's instructions. The results were normalized to the Renilla luciferase corresponding firefly luciferase activities. For each transfection, the luciferase activity was averaged from three replicates.

### Statistical analysis

Data were expressed as mean ± standard deviation (SD) to compare the separate samples. The results were analyzed statistically using one-way analysis of variance or *t*-test. A *P*-value < 0.05 was considered statistically significant. All calculations were performed using the Statistical Program for the Social Sciences (SPSS Inc., Chicago, IL, USA) software 17.0.

## References

[1] Hengge UR, Ruzicka T, Tyring SK, Stuschke M, Roggendorf M, Schwartz RA, Seeber S (2002). Update on Kaposi's sarcoma and other HHV8 associated diseases. Part 1: epidemiology, environmental predispositions, clinical manifestations, and therapy. Lancet Infect Dis.

[2] Kaposi M (1872). Idiopathic multiple pigmented sarcoma of the skin. Arch Dermatol Syphil.

[3] Kaloterakis A, Papasteriades C, Filiotou A, Economidou J, Hadjiyannis S, Stratigos J (1995). HLA in familial and nonfamilial Mediterranean Kaposi's sarcoma in Greece. Tissue Antigens.

[4] Wu XJ, Pu XM, Kang XJ, Halifu Y, An CX, Zhang DZ, Yakeya B, Mijit J (2014). One hundred and five Kaposi sarcoma patients: a clinical study in Xinjiang, Northwest of China. J Eur Acad Dermatol Venereol.

[5] Pu X-M, Wu W-D, Ju H-E (2004). The detection of HHV-8 in the serum of Kaposi's sarcoma before and after the therapy with interferon. Jounal of clinical dermatology.

[6] Wang X, He B, Zhang Z, Liu T, Wang H, Li X, Zhang Q, Lan K, Lu X, Wen H (2010). Human herpesvirus-8 in northwestern China: epidemiology and characterization among blood donors. Virology journal.

[7] Chang Y, Cesarman E, Pessin MS, Lee F, Culpepper J, Knowles DM, Moore PS (1994). Identification of herpesvirus-like DNA sequences in AIDS-associated Kaposi's sarcoma. Science-AAAS-Weekly Paper Edition.

[8] Zhang B, Pan X, Cobb GP, Anderson TA (2007). microRNAs as oncogenes and tumor suppressors. Dev Biol.

[9] Landgraf P, Rusu M, Sheridan R, Sewer A, Iovino N, Aravin A, Pfeffer S, Rice A, Kamphorst AO, Landthaler M, Lin C, Socci ND, Hermida L (2007). A mammalian microRNA expression atlas based on small RNA library sequencing. Cell.

[10] Bartel DP (2009). MicroRNAs: target recognition and regulatory functions. Cell.

[11] O'Hara AJ, Wang L, Dezube BJ, Harrington WJ, Damania B, Dittmer DP. (2009). Tumor suppressor microRNAs are underrepresented in primary effusion lymphoma and Kaposi sarcoma. Blood.

[12] Wu XJ, Pu XM, Zhao ZF, Zhao YN, Kang XJ, Wu WD, Zou YM, Wu CY, Qu YY, Zhang DZ, Feng YY, Liu JY (2015). The expression profiles of microRNAs in Kaposi's sarcoma. Tumour Biol.

[13] Ene AMC, Borze I, Guled M, Costache M, Leen G, Sajin M, Ionica E, Chitu A, Knuutila S (2014). MicroRNA Expression Profiles in Kaposi's sarcoma. Pathology & Oncology Research.

[14] Nikolic I, Plate KH, Schmidt MH (2010). EGFL7 meets miRNA-126: an angiogenesis alliance. J Angiogenes Res.

[15] Fish JE, Santoro MM, Morton SU, Yu S, Yeh RF, Wythe JD, Ivey KN, Bruneau BG, Stainier DY, Srivastava D (2008). miR-126 regulates angiogenic signaling and vascular integrity. Dev Cell.

[16] Li Z, Chen J (2011). *In vitro* functional study of miR-126 in leukemia. Methods Mol Biol.

[17] Liu Y, Zhou Y, Feng X, An P, Quan X, Wang H, Ye S, Yu C, He Y, Luo H (2014). MicroRNA-126 functions as a tumor suppressor in colorectal cancer cells by targeting CXCR4 via the AKT and ERK1/2 signaling pathways. Int J Oncol.

[18] Yang X, Wu H, Ling T (2014). Suppressive effect of microRNA-126 on oral squamous cell carcinoma *in vitro*. Mol Med Rep.

[19] Jusufovic E, Rijavec M, Keser D, Korosec P, Sodja E, Iljazovic E, Radojevic Z, Kosnik M (2012). let-7b and miR-126 are down-regulated in tumor tissue and correlate with microvessel density and survival outcomes in non--small--cell lung cancer. PLoS One.

[20] Foreman KE (2001). Kaposis sarcoma: the role of HHV-8 and HIV-1 in pathogenesis. Expert Rev Mol Med.

[21] Lee RC, Feinbaum RL, Ambros V (1993). The C. elegans heterochronic gene lin-4 encodes small RNAs with antisense complementarity to lin-14. Cell.

[22] Garzon R, Calin GA, Croce CM (2009). MicroRNAs in Cancer. Annu Rev Med.

[23] Ruan K, Fang X, Ouyang G (2009). MicroRNAs: novel regulators in the hallmarks of human cancer. Cancer Lett.

[24] Zhao C, Li Y, Zhang M, Yang Y, Chang L (2015). miR-126 inhibits cell proliferation and induces cell apoptosis of hepatocellular carcinoma cells partially by targeting Sox2. Hum Cell.

[25] Zhu N, Zhang D, Xie H, Zhou Z, Chen H, Hu T, Bai Y, Shen Y, Yuan W, Jing Q, Qin Y (2011). Endothelial-specific intron-derived miR-126 is down-regulated in human breast cancer and targets both VEGFA and PIK3R2. Mol Cell Biochem.

[26] Guo C, Sah JF, Beard L, Willson JK, Markowitz SD, Guda K (2008). The noncoding RNA, miR-126, suppresses the growth of neoplastic cells by targeting phosphatidylinositol 3-kinase signaling and is frequently lost in colon cancers. Genes Chromosomes Cancer.

[27] Liu B, Peng XC, Zheng XL, Wang J, Qin YW (2009). MiR-126 restoration down-regulate VEGF and inhibit the growth of lung cancer cell lines *in vitro* and *in vivo*. Lung Cancer.

[28] Zhang J, Du YY, Lin YF, Chen YT, Yang L, Wang HJ, Ma D (2008). The cell growth suppressor, mir-126, targets IRS-1. Biochem Biophys Res Commun.

[29] Lee J, Jung ID, Chang WK, Park CG, Cho DY, Shin EY, Seo DW, Kim YK, Lee HW, Han JW, Lee HY (2005). p85 beta-PIX is required for cell motility through phosphorylations of focal adhesion kinase and p38 MAP kinase. Exp Cell Res.

[30] Foukas LC, Berenjeno IM, Gray A, Khwaja A, Vanhaesebroeck B (2010). Activity of any class IA PI3K isoform can sustain cell proliferation and survival. Proc Natl Acad Sci U S A.

[31] Ueki K, Fruman DA, Yballe CM, Fasshauer M, Klein J, Asano T, Cantley LC, Kahn CR (2003). Positive and negative roles of p85 alpha and p85 beta regulatory subunits of phosphoinositide 3-kinase in insulin signaling. J Biol Chem.

[32] Zhang Z, Zhang T, Zhou Y, Wei X, Zhu J, Zhang J, Wang C (2015). Activated phosphatidylinositol 3-kinase/Akt inhibits the transition of endothelial progenitor cells to mesenchymal cells by regulating the forkhead box subgroup O-3a signaling. Cell Physiol Biochem.

[33] Cortes I, Sanchez-Ruiz J, Zuluaga S, Calvanese V, Marques M, Hernandez C, Rivera T, Kremer L, Gonzalez-Garcia A, Carrera AC (2012). p85beta phosphoinositide 3-kinase subunit regulates tumor progression. Proc Natl Acad Sci USA.

[34] Siegal B, Levinton-Kriss S, Schiffer A, Sayar J, Engelberg I, Vonsover A, Ramon Y, Rubinstein E (1990). Kaposi's sarcoma in immunosuppression. Possibly the result of a dual viral infection. Cancer.

